# Nonlinear analysis of heart rhythm in preeclampsia: a route for translational clinical applications in neuroinflammation

**DOI:** 10.1186/s40885-021-00182-2

**Published:** 2021-12-15

**Authors:** José Javier Reyes-Lagos, Eric Alonso Abarca-Castro

**Affiliations:** 1 Autonomous University of the State of Mexico (UAEMex), School of Medicine, Toluca, State of Mexico Mexico; 2 Autonomous University of the State of Mexico (UAEMex), Multidisciplinary Research Center in Education (CIME), Toluca, State of Mexico Mexico

**Keywords:** Pregnancy, heart rate, Neuroimmunomodulation, Pre-eclampsia, Autonomic nervous system

## Abstract

Preeclampsia is a pregnancy-specific condition which gets detected through hypertension and excessive protein excretion in urine. While preeclampsia used to be regarded as a self-limiting maternal condition which resolved with the delivery of the placenta, it is nowadays considered a complex and multifactorial disease that affects the offspring. Unfortunately, the etiology and pathophysiology of this multifaceted disorder remain elusive. Recent findings have confirmed that an altered maternal autonomic function may play a vital role in developing preeclampsia in conjunction with an imbalanced maternal immune system. Additionally, further evidence supports the crucial role of an exacerbated immune response driven by a non-infectious trigger during preeclampsia. Therefore, as a sterile inflammation, the elucidation of the neuroinflammatory mechanisms of preeclampsia warrants obtaining relevant knowledge suitable for translational clinical applications.

Heart rate variability (HRV) is an affordable and non-invasive method for indirectly assessing the autonomic nervous system and the cholinergic anti-inflammatory pathway (CAP). Notably, the nonlinear analysis of HRV offers novel indexes to explore the neuroimmune interactions in diverse preclinical and clinical settings of inflammation. Given that the dynamics of HRV is nonlinear in health, we hypothesized that a neuroinflammatory condition in preeclampsia might be associated with changes in nonlinear features of maternal and fetal HRV. Thus, the present review aims to present evidence of the potential changes in maternal-fetal HRV associated with neuroinflammatory modifications in preeclamptic women. We considered that there is still a need for assessing the nonlinear features of maternal and fetal HRV as complementary biomarkers of inflammation in this population in future studies, being a potential route for translational clinical applications.

## Background

Preeclampsia is one of the leading causes of critical maternal and fetal mortality and morbidity. It is diagnosed by pregnancy-onset hypertension and proteinuria after 20 weeks of gestation [1]. Both preeclamptic women and their newborns are at higher risk of higher risk of developing severe cardiovascular complications and metabolic syndromes later in life [2].

Preeclampsia’s prevalence is estimated to be seven times higher in low-income countries compared to high-income countries [2]; thus, it is a public health problem and a significant cause of maternal death. The 2030 Agenda for Sustainable Development and the Millennium Development Goals have placed maternal well-being as a top priority concern, and the corresponding goal 3 is to decrease by 2030 the global maternal mortality ratio below 70 per 100,000 live births [3, 4]. In Mexico, the current maternal mortality ratio is estimated as 33 per 100,000 live births [5]. According to the World Health Organization (WHO), the most common direct causes of maternal mortality are preeclampsia/eclampsia, obstetric hemorrhage, and puerperal sepsis [6]. Authors suggest that a decrease in maternal mortality rate in low-income countries is associated with Human Development Index; thus, by increasing educational levels, income per capita, and life expectancy, a substantial reduction in maternal and child mortality rates is observed [7].

Conventionally, preeclampsia has been regarded as a self-limiting maternal pathological condition which gets completely resolved with the placenta’s delivery. However, it is also considered a complex and multifactorial disease that may also have consequences for the offspring [8]. It is known that some characteristics of preeclampsia are: placental oxygen dysregulation, abnormal trophoblast invasion, overexpression of anti-angiogenic factors, anomalous maternal-fetal immune interactions [8], maternal autonomic dysfunction [9], and distress conditions [10].

Preeclampsia remains a particular disorder of pregnancy, with an incomplete understanding of its etiology [11]. Recent findings confirmed that an altered maternal autonomic function could play a key role in developing preeclampsia in conjunction with an imbalanced immune system [12, 13]. It is noteworthy to mention that some clinical studies have demonstrated that this immune imbalance induced by preeclampsia promotes a chronic inflammatory state during pregnancy [14]. Additionally, authors have described preeclampsia as a multisystem disorder based on a cascade of immunopathological events originating from the placenta [15]. However, it is recognized that a single candidate mechanism does not explain preeclampsia’s complex pathogenesis [15].

New disciplines such as neuroimmunology have emerged in order to provide a compelling integrative link between the immune system and nervous system. It recognizes that immune responses are present in the central and peripheral nervous systems during disease, considering the existence of a neuroimmune axis [16]. Some authors have proposed that novel psychophysiological indexes can assess the neurophysiological pathway responsible for adaptively regulating inflammatory processes in humans [17]. For example, the analysis of heartbeat fluctuations or heart rate variability (HRV) is a convenient, non-invasively, and economical method for quantifying the cardiac autonomic nervous system (ANS) [18]. In addition, the HRV has recently been considered an important “window” to understand better the neuroimmune system involved in inflammatory conditions [19, 20]. Studies even reveal that several indexes of HRV seem to be related to inflammatory biomarkers in humans [21–24]. The exploration of HRV has provided a deeper understanding of the cholinergic anti-inflammatory pathway (CAP) [25–28], a neuroimmune mechanism that inhibits pro-inflammatory cytokine release via the vagus nerve [29].

According to the literature, linear approaches of HRV have been extensively studied and are not adequate to fully describe a complex system [30]. Particularly, nonlinear features of HRV are becoming increasingly important in present and future studies to comprehend complex biological systems in both health and disease [31]. Given that the healthy HRV time series shows nonlinear features, we hypothesized that a neuroinflammatory condition in preeclampsia might be associated with changes in nonlinear features of maternal and fetal HRV.

This review article aims to present evidence of the potential changes in HRV associated with neuroinflammatory modifications in preeclamptic women; we first describe the immune and neuroimmune profiles in preeclampsia, followed by linear and nonlinear HRV changes during preeclampsia. Finally, we discuss the nonlinear analysis of HRV as a novel tool for exploring inflammatory mechanisms in the maternal-fetal dyad during preeclampsia.

### Immune and neuroimmune profiles in preeclampsia

According to some studies, the immune profile of women with preeclampsia shifts from a steady state of mild inflammation; from an equilibrium of both pro-inflammatory and anti-inflammatory mechanisms to a downregulated state characterized by increased inflammatory cytokines and effector immune cells with a concomitant decrease in anti-inflammatory factors and regulatory cells [32]. Other recent findings have documented an increased ratio of pro- to anti-inflammatory cytokines in preeclampsia, suggesting that the decreased levels of cytokines such as interleukin 4 (IL-4) and interleukin 10 (IL-10) accelerate the production of pro-inflammatory cytokines resulting in excessive inflammation [33]. Interestingly, further experimental studies showed that by infusing IL-10 to restore the immune system’s balance, the blood pressure associated with placental ischemia in pregnant rats is reduced [13]. These results support a relevant role of the anti-inflammatory cytokines in preeclampsia. In line with such findings, novel studies report an association between the pro-inflammatory cytokine profile and lower plasma concentrations of soluble CD163 molecule and IL-10 of women with severe preeclampsia [34]. Authors have thus endorsed that assessing the cytokine profile of women with preeclampsia is convenient [35].

**Fig. 1:**
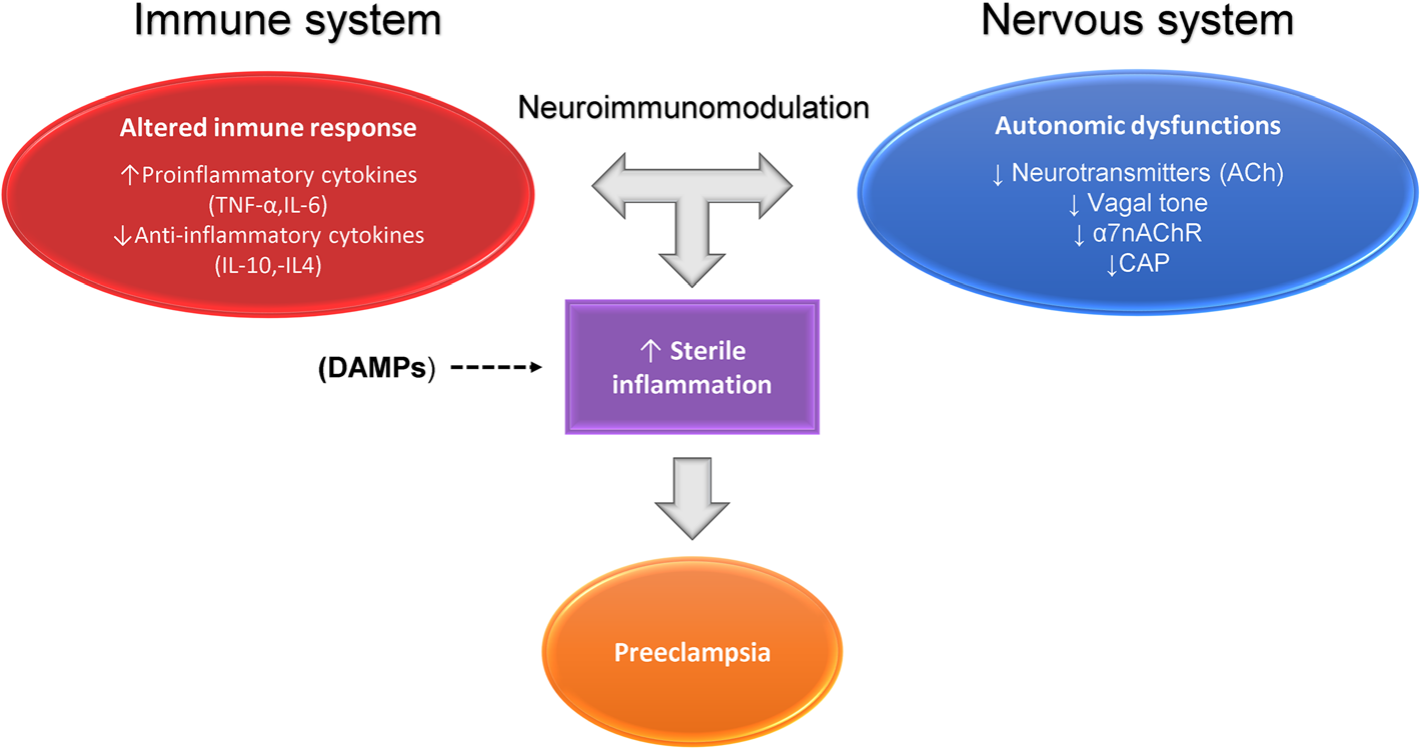
Preeclampsia etiology or exacerbation may be mediated by an upregulated sterile inflammation, it may be initiated by molecules in the host organism known as damage associated molecular patterns (DAMPs). In addition to DAMPs, the upregulation of this sterile inflammation is produced by an altered immune response in conjunction with autonomic dysfunctions. Particularly, immune alterations are characterized by the release of pro-inflammatory cytokines such as tumor necrosis factor-alpha (TNF-α) and interleukin-6 (IL-6) and decrease of anti-inflammatory cytokines such as interleukin-4 (IL-4) and interleukin-10 (IL-10). Finally, autonomic dysfunctions may involve diminished acetylcholine (ACh) synthesis, lower vagal tone, downregulation of both alpha 7 nicotinic acetylcholine receptor (α7nAChR) and cholinergic anti-inflammatory pathway (CAP)

Preclinical evidence has indicated that interleukin 6 (IL-6) may play a role in mediating hypertension and reducing renal hemodynamics observed in uterine perfusion reductions in pregnant rats [36]. According to Conrad et al., elevated levels of tumor necrosis factor-alpha (TNF-α) and IL-6 may contribute to preeclampsia’s putative endothelial dysfunction [37]. Novel evidence confirms that high levels of TNF-α have been associated with an increased risk of preeclampsia [38]. Additionally, new findings support a non-bacterial maternal immune response role in the development of preeclampsia and eclampsia [39]. For example, a sterile inflammation may be initiated by molecules in the host organism known as damage associated molecular patterns (DAMPs), Fig. 1. In preeclampsia, various DAMPs may be involved in this disorder’s etiology or exacerbation [39].

Yang et al. [40] reported reduced vagus nerve function in preeclamptic women. Consequently, in these women, evidence suggests that diminished acetylcholine (ACh) synthesis may contribute to excessive inflammation and hypertension [41]. ACh serves as a neuromodulator in the central nervous system, affecting neuronal excitability, synaptic transmission, and plasticity [41]. Findings show that the mRNA expression levels of the alpha 7 nicotinic acetylcholine receptor (α7nAChR) in preeclamptic women’s monocytes were lower than control healthy women’s monocytes. Thus, the authors concluded that the downregulation of α7nAChR may be linked to the development of preeclampsia by boosting pro-inflammatory and decreasing anti-inflammatory cytokine release via the nuclear factor kappa β (NF-κβ) pathway [42]. Considering its ability to modulate anti-inflammatory processes by reducing the expression of pro-inflammatory effectors and cytokines, α7nAChR has received much attention in the last few decades [43]. Therefore, preeclampsia can be considered an altered neuroimmune condition in pregnant women, becoming a frontier topic for multiple disciplines, Fig. 1.

Novel findings suggest that the vagal-driven activation of the α7nAChR attenuates preeclampsia-like symptoms, and this protective effect is likely result of the inhibition of inflammation via the NF-κβ pathway [44].

McAllen et al., have provided compelling evidence in preclinical studies that indicates that splanchnic sympathetic outflow acts to suppress immune-mediated inflammation [45]. However, recent evidence shows that preeclampsia is not associated with elevated sympathetic reactivity evaluated by sympathetic nervous system activity [46]. Consistently, current evidence suggests that an autonomic dysfunction is highly prevalent in preeclamptic women [12].

There is still a lack of knowledge in how preeclampsia modifies the neuroimmune pathways; for example, some studies have reported an association among maternal immune activation, the manifestation of preeclampsia, and an altered neonatal neurodevelopment [47]. A possible pathway for this association involves the dysfunction of the microglial cells, which are the brain’s immune cells [47]. In this sense, studies are necessary to provide more apprehensible knowledge about the neuroinflammatory mechanisms in preeclampsia. Biomedical research is pushing forward to developing potential non-invasive and fast diagnostic biomarkers for clinical translational applications.

### Linear and nonlinear HRV changes during preeclampsia

Nonlinear HRV analysis is based on chaos theory, in which the heart rhythm generation, besides being sensitive to initial conditions and evolving very fast over time, is considered nonlinear [48, 49]. On the other hand, when using linear analysis techniques of HRV data, several assumptions must be made to ensure that the data is meaningfully interpreted. For example, the HRV data must be stationary, i.e., the mean and variance must be stable. However, it is recognized that the cardiac system is dynamic, nonlinear, and nonstationary [49]. Thus, linear methods of HRV may not account for all aspects of cardiac performance, especially the complex interactions between the control mechanisms that regulate heart function, due to assumptions and conditioning needed for linear HRV analysis [49]. In any case, both linear and nonlinear processing algorithms of HRV have provided useful computational tools for diagnosing a wide range of inflammatory conditions and pathologies [26–29]. According to a review of 2020, the most measurable nonlinear features of HRV are representation features (e.g., Poincaré plot representation, recurrence plot analysis, asymmetry); fractal (e.g., detrended fluctuation analysis, Hurst exponent); entropy or complexity-based features (e.g., Shannon entropy, sample entropy, multiscale entropy) and symbolic dynamics [50].

Evidence has also shown negative associations between linear HRV indexes such as the standard deviation of the normal-to-normal interval with TNF-α in heart failure patients, suggesting that the immune system’s activation is related to autonomic imbalance [51]. More specifically, our previous research indicates that nonlinear features of HRV such as entropy-based methods, symbolic dynamics, and fractal methods have successfully characterized systemic acute inflammatory conditions such as the lipopolysaccharide (LPS) induced endotoxemia [52–54] and low-risk human parturition at term [55].

Specifically, it is known that preeclampsia affects the cardiovascular health of both mother and offspring [56]. Studies also indicated that different hypertensive disorders such as chronic hypertension, preeclampsia, and pregnancy-induced hypertension (PIH) are different in HRV, indicating that different regulatory mechanisms are involved [57]. In Mexican women, PIH’s progression into preeclampsia has been appreciated in one of each four patients [35]; thus, the evaluation of HRV may be considered an additional biomarker for monitoring PIH patients who may develop preeclampsia. A recent review study indicates that, in general, a decrease in overall HRV is found in preeclampsia compared to normotensive pregnant controls [58]. Among different autonomic alterations in preeclampsia, a reduction in the autonomic vagal modulation and increased sympathetic autonomic modulation assessed by HRV analysis have been reported [9, 40].

Novel nonlinear measures have been introduced to describe HRV, which present the advantage of not being affected by nonstationary effects [59]. Table 1 summarizes the primary outcomes of clinical and preclinical studies of HRV and preeclampsia [9, 40, 57, 60–66]. Table 1 specifies the type of HRV measure (linear or nonlinear), and if the analysis was performed in fetal or maternal HRV data. Interestingly, only one (1/10) of the consulted studies have evaluated nonlinear analysis of maternal or fetal HRV in women with preeclampsia.

**Table 1:** Study summaries of heart rate variability and preeclampsia

Study	Sample size	Monitoring method	HRV measure	Conclusions
Clinical: maternal Chaswal et al. (2018 )[9]	120	HRV	Linear	A reduction in autonomic vagal modulation and an increase in sympathetic autonomic modulation in preeclampsia.
Clinical: maternal Yang et al. (2000 )[40]	45	HRV	Linear	Preeclampsia is associated with a facilitation of sympathetic regulation and attenuation of parasympathetic influence of heart rate.
Clinical: maternal Faber et al. (2004 )[57]	161	HRV	Linear Nonlinear	Parameters of the HRV differ between various hypertensive pregnancy disorders.
Preclinical: fetal Abuiessa et al. (2020 )[60]	64	HRV	Linear	Preeclampsia accentuates endotoxic manifestations of hypotension, tachycardia, and cardiac autonomic dysfunction in male offspring.
Clinical: maternal Speranza et al. (2019 )[61]	60	HRV	Linear	Autonomic activity increases during postpartum in preeclamptic women with severe features.
Clinical: maternal and fetal Lakhno. (2017 )[62]	106	HRV Fetal CTG	Linear	The maternal and fetal hemodynamic coupling was reduced in preeclampsia.
Clinical: maternal and fetal Hoyer et al. (2017 )[63]	106	HRV	Linear	A sympathetic overactivity and the lack of vagal regulation lead to the loss of link between maternal and fetal correlations of heart rate patterns in severe preeclampsia.
Clinical: maternal Hossen et al. (2017 )[64]	40	HRV	Linear	During pregnancy and preeclampsia, parasympathetic activities are reduced, and sympathetic activities are increased compared to healthy women.
Clinical: maternal Musa et al. (2016 )[65]	120	HRV	Linear	A dominant cardiac sympathetic modulation on patients with preeclampsia was found, probably secondary to parasympathetic withdrawal.
Clinical: fetal Lakhno. (2014 )[66]	160	HRV	Linear	A decreased fetal autonomic tone and predominance sympathetic regulation was marked in preeclampsia.

*HRV* heart rate variability, *CTG* cardiotocography

HRV extracted from the maternal or fetal electrocardiogram

### Nonlinear measures of HRV: a novel tool for the exploration of the CAP

Nonlinear HRV measures or features have shown potential as biomarkers of internalizing psychopathology, often associating lower information-based complexity with internalizing psychopathology [67]. The relationships between physiological variability, interorgan communication, and disease suggest that monitoring complexity may reveal disease state; thus, decreased complexity would correspond with disease progression, and increased complexity (towards homeostasis) would correspond with recovery [68]. The complexity analysis of HRV data, which also quantifies nonlinear interactions among frequencies reflecting underlying ANS dynamics, represents a relevant topic in assessing neuroimmune disorders. However, this complexity also means that there is potentially a variety of information from a wide range of physiological systems embedded in the HRV, indicating an opportunity to utilize computational approaches to reveal the physiological significance of HRV.

Nonlinear measures of HRV have already allowed the discrimination of depressive patients from healthy subjects, consistently showing a significant decrease of HRV complexity in neuroimmune pathologies [69]. Mainly, multiscale entropy, a complexity measure of HRV, was more sensitive than current clinical markers for evaluating inflammation in a preclinical model of LPS-induced inflammation [70]. Authors suggest that multiscale entropy may prove a fruitful component of a bedside monitor to detect inflammatory insults.

According to the consulted literature, no clinical studies have been conducted to simultaneously evaluate the nonlinear features of HRV and multiple inflammation markers in women diagnosed with preeclampsia. However, results indicate that preeclamptic women reveal a reduced complexity by incrementing periodicity assessed by multiscale asymmetry indexes of HRV [71]. The authors concluded that the mathematical indexes obtained with the nonlinear methods of HRV could differentiate between normal and preeclamptic pregnancies. At present, there is a clinical need to diagnose preeclampsia accurately and promptly, which usually progresses to an adverse fetal or maternal outcome.

**Fig. 2:**
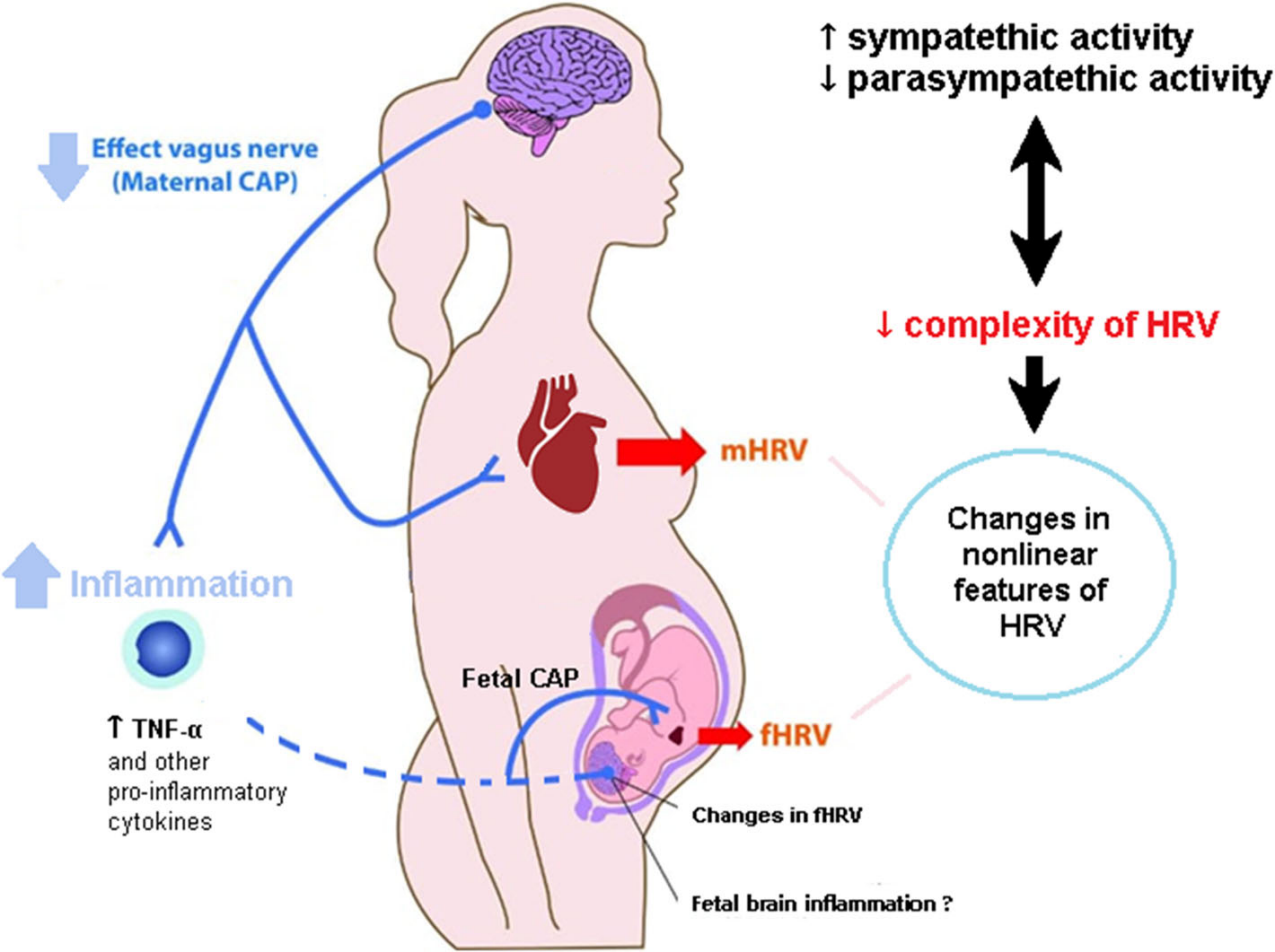
Alterations in preeclampsia may involve increased cardiac sympathetic activity, decreased cardiac parasympathetic activity, and reduced complexity of heart rate fluctuations. These alterations may produce changes in nonlinear features of the maternal or fetal heart rate variability (mHRV and fHRV, respectively). A downregulated maternal cholinergic anti-inflammatory pathway (CAP) may result in a high pro-inflammatory state in the mother and fetus (increased levels of tumor necrosis factor-alpha [TNF-α], interleukin-6 [IL-6], and other pro-inflammatory cytokines). The assessment of nonlinear features of heart rate variability (HRV) is a potential tool for exploring the neuroinflammatory interactions in the maternal-fetal dyad

The CAP activity can be monitored easily and non-invasively via the linear analysis of HRV data derived from either maternal or fetal electrocardiogram, Fig. 2. However, the linear analysis of HRV is not entirely appropriate to offer information about the complex dynamics of heartbeat fluctuations, and consequently, from the CAP activity. This is because the mechanisms involved in cardiovascular physiology interact with each other in a nonlinear way [72]. The activation of the CAP could rather be crucial for controlling preeclampsia symptoms. Besides, nonlinear methods of HRV not only present clinical relevance but also offer an improved interpretation of pathological conditions. Additionally, the main advantage of HRV signals is that they can be calculated in real-time and non-invasively, while all current biomarkers used in clinical practice are discrete and imply blood sample analyses which delay results.

The information derived from chaos theory, fractal mathematics, and the dynamic complexity of HRV has not yet been fully applied in the obstetrics field. It is a productive area for research in both healthy and pathological scenarios. Also, the complex characterization of the HRV time series during preeclampsia has rarely been studied. Theoretically, the maternal and fetal nonlinear analysis of HRV offers unique opportunities to explore the neuroimmune interactions in the mother-child binomial.

## Conclusions

Neuroinflammatory alterations in preeclamptic women may involve autonomic changes associated with a possible concomitant downregulated CAP and modifications in nonlinear features of HRV. We consider that the assessment of nonlinear measures of both maternal and fetal HRV during preeclampsia is a promising field for exploring the neuroinflammatory interactions in the maternal-fetal dyad and a route for potential translational clinical applications. The knowledge of this frontier topic promotes basic research and translational clinical applications for scientists worldwide who conduct research concerning inflammatory processes.

## Data Availability

Not applicable.

## Abbreviations

α7nAChR: Alpha 7 nicotinic acetylcholine receptor
ACh: Acetylcholine
ANS: Autonomic nervous system
CAP: Cholinergic anti-inflammatory pathway
DAMPs: Damage associated molecular patterns
HRV: Heart rate variability
IL-4: Interleukin 4
IL-6: Interleukin 6
IL-10: Interleukin 10
LPS: Lipopolysaccharide
NF-κβ: Nuclear factor kappa β
PIH: Pregnancy-induced hypertension
TNF-α: Tumor necrosis factor-alpha
WHO: World Health Organization
